# The Impact of N-Acetyl Cysteine and Coenzyme Q10 Supplementation on Skeletal Muscle Antioxidants and Proteome in Fit Thoroughbred Horses

**DOI:** 10.3390/antiox10111739

**Published:** 2021-10-30

**Authors:** Marisa L. Henry, Deborah Velez-Irizarry, Joe D. Pagan, Lorraine Sordillo, Jeff Gandy, Stephanie J. Valberg

**Affiliations:** 1 Department of Large Animal Clinical Sciences, College of Veterinary Medicine, Michigan State University, East Lansing, MI 48824, USA; velezdeb@msu.edu (D.V.-I.); Sordillo@msu.edu (L.S.); gandy@msu.edu (J.G.); valbergs@msu.edu (S.J.V.); 2 Kentucky Equine Research, Versailles, KY 40383, USA; pagan@ker.com

**Keywords:** antioxidants, glycolysis, mitochondria, mitochondrial proteins, reactive oxygen species, redox

## Abstract

Horses have one of the highest skeletal muscle oxidative capacities amongst mammals, which, combined with a high glycolytic capacity, could perturb redox status during maximal exercise. We determined the effect of 30 d of oral coenzyme Q10 and N-acetyl-cysteine supplementation (NACQ) on muscle glutathione (GSH), cysteine, ROS, and coenzyme Q10 concentrations, and the muscle proteome, in seven maximally exercising Thoroughbred horses using a placebo and randomized cross-over design. Gluteal muscle biopsies were obtained the day before and 1 h after maximal exercise. Concentrations of GSH, cysteine, coenzyme Q10, and ROS were measured, and citrate synthase, glutathione peroxidase, and superoxide dismutase activities analyzed. GSH increased significantly 1 h post-exercise in the NACQ group (*p* = 0.022), whereas other antioxidant concentrations/activities were unchanged. TMT proteomic analysis revealed 40 differentially expressed proteins with NACQ out of 387 identified, including upregulation of 13 mitochondrial proteins (TCA cycle and NADPH production), 4 Z-disc proteins, and down regulation of 9 glycolytic proteins. NACQ supplementation significantly impacted muscle redox capacity after intense exercise by enhancing muscle glutathione concentrations and increasing expression of proteins involved in the uptake of glutathione into mitochondria and the NAPDH-associated reduction of oxidized glutathione, without any evident detrimental effects on performance.

## 1. Introduction

Horses have one of the highest skeletal muscle oxidative capacities amongst mammals, with evolutionary adaptations in heart size and lung capacity maximizing aerobic metabolism [1]. During aerobic muscle contraction, both ATP and reactive oxygen species (ROS) are produced in the mitochondria. Physiological amounts of ROS generated in skeletal muscle serve important adaptive signaling functions to prevent muscle fatigue, however, excessive ROS can overwhelm antioxidant capacity, causing oxidative damage to proteins, lipids, and DNA, with detrimental effects on muscle function [2]. Both nonenzymatic and enzymatic cellular antioxidants reduce ROS. Glutathione (γ-l-glutamyl-l-cysteinylglycine) is the most abundant nonenzymatic antioxidant in mammalian cells [3]. It maintains the thiol status of critical proteins via cysteine’s sulfhydryl group and defends against ROS via its reducing capacity [4]. Glutathione is synthesized endogenously from glutamate, cysteine, and glycine with cysteine availability being the rate-limiting step in synthesis [5]. Mammalian cells do not have a large storage pool of free cysteine, rather, cysteine is either synthesized from methionine [5] or circulated to skeletal muscle in its oxidized form, cystine, where it is transported into the myoplasm and reduced to cysteine [6].

Enhancing plasma or tissue glutathione concentrations can be accomplished in other species, including humans, by increasing the availability of cysteine [7]. Human studies have utilized N-acetylcysteine (NAC) because cysteine is easily oxidized, has a relatively unstable shelf life, and its disulfide cystine is poorly soluble in water, affecting absorption [7,8]. NAC increases the unbound pool of circulating cysteine and cells exhibit high transport activity for this reduced form, whereas transport activity for the larger disulfide cystine is relatively low [9]. One study supplemented horses with oral NAC at 20 mg/kg and showed that NAC has an anti-inflammatory effect in the endometrium [10]. Although muscle concentrations or activities of antioxidants such as vitamin E [11,12,13] have been studied in supplementation trials, concentrations of skeletal muscle glutathione and many other antioxidants have not been reported in horses.

Ubiquinone, also known as coenzyme Q10, (CoQ10; 2,3 dimethoxy-5 methyl-6-decaprenyl benzoquinone) is another potent cellular antioxidant. CoQ10 serves an additional essential role in the mitochondrial electron transport system where it accepts electrons from complex I (NADH ubiquinone oxidoreductase) and complex II (succinate ubiquinone reductase) and transfers them to complex III (ubiquinol cytochrome c reductase) [14]. CoQ10 supplementation has been recommended for human athletes and for treatment of myopathies, CoQ10 deficiencies, and neurodegenerative and cardiovascular disease in humans [14]. In horses, plasma CoQ10 concentrations have been shown to increase with oral supplementation of 1–3.4 g/d [15,16]. Only one study has evaluated muscle CoQ10 concentrations in horses [17]. In that study, fit Thoroughbred horses supplemented for 21 d with 1 g/d of oral CoQ10 had significantly increased gluteal muscle CoQ10 concentrations [17].

As elite athletes, Thoroughbred racehorses have maximal oxygen consumption that is two to three times greater than elite human athletes and a higher mitochondrial mass than many other species [18,19]. Thus, it seems likely that Thoroughbred horses produce large amounts of ROS during maximal exercise, which could have detrimental physiological impacts if ROS overwhelm antioxidant capacity. High levels of ROS have been measured in equine cultured myoblasts during hypoxia but not in skeletal muscle biopsies, to the authors’ knowledge [20]. Enhancing the most ubiquitous antioxidant, glutathione, and the most potent antioxidant, CoQ10, in skeletal muscle could have beneficial effects on skeletal muscle in equine athletes and horses with myopathies impacted by oxidative stress [21,22].

The purpose of the present study was to determine the effect of oral supplementation of NAC and CoQ10 (NACQ) on skeletal muscle ROS and enzymatic and nonenzymatic antioxidant concentrations/activities in exercising Thoroughbred horses. A second objective was to determine if NACQ supplementation had an impact on the skeletal muscle proteome.

## 2. Materials and Methods

Horses: seven Thoroughbred horses (five geldings and two mares, 4.7 ± 2.1 y) were galloped over 1½ miles on the track 3 d/wk and walked (10 min) and trotted (20 min) on an automated exercise machine 3 d/wk. Horses were turned out for 3–6 h/d in a grass paddock for 3 d/wk on exercise days and 8 h/d on their rest day. Throughout the trial, horses were fed timothy hay ad libitum and 2.1 kg/500 kg body weight of concentrate 3 times/d on exercise days and 2–3 times/d on rest days (OBS Racing Blend, Ocala Breeders Feed & Supply Ocala, FL) (Table 1). The amount fed was adjusted based on weekly body weights.

Study design: a randomized cross-over design was used where horses were supplemented with a placebo (40 g of an electrolyte supplement containing 9.6 g sodium, 2 g potassium, 16 g chloride, 3.6 g calcium, and 1.2 g magnesium (Race Recovery, KER) once/d) or NACQ for 30 d. Daily NACQ consisted of 1.6 g CoQ10 (Nano Q10, KER Versailles, KY, USA), 10 g NAC, and the same electrolyte supplement as the placebo. The dose of CoQ10 and NAC was divided into the morning and evening concentrate and horses were watched to ensure complete consumption.

Test protocol: on Day 30 and Day 60, horses performed an exercise test consisting of a ½ mile (0.8 km) breeze on the track at the horse’s top speed while wearing a heart rate monitor with GPS (KER Clockit Polar monitor). During the second exercise test on Day 60, the same experienced rider was instructed to replicate the GPS-determined speed from the Day 30 exercise test.

Muscle samples: the day prior to the exercise test (Day 29, 59), horses were sedated with 200 mg xylazine IV and a percutaneous needle biopsy of the gluteus medius muscle was obtained from a standardized site 16 cm along a line from the highest point of the tuber coxae to the tail head using local anesthesia [23]. The muscle biopsy procedure was repeated using the alternate side 1 h after the exercise test on Days 30 and 60. Muscle samples were immediately flash-frozen in liquid nitrogen.

Blood samples: jugular venous blood samples were obtained prior to the exercise test and 10 min, 1 h, and 4 h after the exercise test. A buffy coat from EDTA tubes was obtained from each horse and frozen at −80 °C. Plasma samples were kept chilled on ice or in the refrigerator, centrifuged within 2 h of collection, frozen on dry ice, and shipped with muscle samples to the laboratory on dry ice where they were stored at −80 °C until analyses.

## 3. Analyses

### 3.1. Blood Samples

Plasma lactate concentrations were analyzed using a YSI lactate analyzer (YSI incorporated, Yellow Springs, OH, USA). Plasma creatine kinase (CK) and gamma-glutamyl transferase (GGT) activity was assayed in pre-exercise and 4 h-post-exercise samples at the Veterinary Diagnostics Laboratory at Michigan State University using standard methods.

DNA was isolated from 250 μL buffy coat using the Qiagen DNA extraction kit (Qiagen, Hilden, Germany). Myostatin genotypes were determined using primers designed to amplify a 150 bp region containing a previously identified single-nucleotide polymorphism (g.66,493,737C>T) as previously described [24,25]. Sanger sequencing and Sequencher software (Gene Codes Corporation, Ann Arbor, MI, USA) were used to determine myostatin genotypes.

### 3.2. Muscle Biochemistry

Muscle homogenate: for the ROS, cysteine, and glutathione assays, approximately 30 mg of muscle was homogenized in radioimmunoprecipitation assay buffer (RIPA). Protein content of the homogenate was determined using a Pierce BCA assay kit (Thermo Scientific, Waltham, MA, USA).

Reactive oxygen species: ROS were analyzed in resting and post-exercise muscle samples using 10 μL of the homogenate diluted 20x in RIPA buffer. An oxidant-sensitive fluorescent probe kit (OxiSelect STA-347, Cell BioLabs, San Diego, CA, USA) was used to measure total ROS, which included reactive nitrogen species, including hydrogen peroxide, nitric oxide, peroxyl radical, and peroxynitrite anion in resting and 1 h-post-exercise muscle samples. Sample concentrations were measured using a Synergy h1 plate reader (Biotek, Winooski, VT, USA) with 480 nm excitation and 530 nm emission in a 96-well black plate with white wells. All samples and standards were measured in triplicate and concentrations were measured as hydrogen peroxide (H_2_O_2_) equivalence.

Thiols: cysteine and glutathione concentrations were analyzed in resting and post-exercise muscle homogenates using liquid chromatography tandem mass spectrometry (LC/MS). Protein in the homogenate was reduced using Tris(2-carboxyethyl)phosphine hydrochloride in water (pH 7) containing N-ethylmaleimide which binds to the active sulfhydryl group, stopping any subsequent reactions. An internal standard of 20 μM GSH ammonium salts D-5 (Toronto Research Chemicals, Toronto, ON, Canada) was added to all samples and standards. Chromatographic separation of cysteine and glutathione was achieved using a Phenomenex Kinetex 1.7 μm EVO C18 100A (50 × 2.1 mm) (Phenomenex, Torrance, CA, USA) column with a gradient elution consisting of acetonitrile and deionized water with 0.1% formic acid. Multiple reaction monitoring, optimized using Waters Empower 4 software, was used for detection of ions generated by cysteine and glutathione in the LC/MS.

CoQ10:CoQ10 analysis of resting muscle samples was performed at the Michigan State University Mass Spectrometry and Metabolomics Core using a high-resolution/accurate-mass (HR/AM) UHPLC-MS/MS system consisting of a Thermo Vanquish UHPLC interfaced with Thermo Q-Exactive (Thermo Fisher Scientific, Waltham, MA, USA) according to Pandey 2018 [26]. Approximately 10 mg of tissue was homogenized in a 95:5 ethanol:2-propanol solution containing 500 ng/mL CoQ4 internal standard with 125 μg of butylated hydroxytoluene pre-dried in the homogenization tube. CoQ10 was extracted from this homogenate by adding 400 μL of hexane then 200 μL milli-Q water. The organic phase was collected, evaporated, and then reconstituted in 2 mL of ethanol containing 0.3 M hydrochloric acid. A 10 μL amount of sample was injected onto a Waters Acquity BEH-C18 UPLC column (2.1 × 100 mm, Waters Corporation, Milford, MA, USA) and eluted using a 5 min isocratic flow of 5 mM ammonium formate in 2-propanol/methanol (60:40 *v*/*v*) at 0.3 mL/min. Compounds were ionized by electrospray operating in positive ion mode with a spray voltage of 3.5 kV, capillary temperature of 256.25 °C, probe heater temperature of 412.50 °C, and S-Lens RF level of 50. Spectra were acquired using a full MS/all-ion fragmentation method at 70,000 resolution, AGC target of 1.0 × 10^6^ and mass range of *m*/*z* 150–1000. The normalized collision energy for the AIF scans was set to 22 V. Data were processed using Xcalibur software version 4.1.31.9.

Glutathione Peroxidase and Superoxide Dismutase: glutathione peroxidase and superoxide dismutase activities were measured in resting and post-exercise muscle samples using colorimetric assay kits (Cayman Chemical, Ann Arbor, MI, USA) according to manufacturer’s instructions. For the superoxide dismutase assay, 10 mg of tissue was homogenized in pH 7.2 buffer containing 20 mM 2-[4-(2-hydroxyethyl)piperazine-1-yl]ethanesulfonic acid, 1 mM ethylene glycol-bis(β-aminoethyl ether)-N,N,N′,N′-tetraacetic acid, 210 mM mannitol, and 70 mM sucrose. For the glutathione peroxidase assay, 10 mg of tissue was homogenized in pH 7.5 buffer containing 50 mM Tris-HCl, 5 mM ethylenediaminetetraacetic acid, and 1 mM dithiothreitol. The reducing agent BCA assay kit (Thermo Fisher Scientific, Waltham, MA, USA) was used to determine protein content for glutathione peroxidase and superoxide dismutase homogenates.

Citrate Synthase: citrate synthase (CS) activity, a marker for mitochondrial volume density, was assayed in resting muscle samples. Approximately 10 mg of gluteal muscle was homogenized in 0.1 M phosphate buffer (pH 7.3) and the activity of citrate synthase was determined fluorometrically at 25 °C according to Essen and Henrickson et al., 1984 [27].

### 3.3. Statistical Analysis

Data were analyzed for normality using a Shapiro–Wilks normality test. Maximal heart rates during the exercise test were compared between placebo and NACQ using a paired *t*-test. Plasma lactate concentrations, GGT, and CK activities were analyzed using a repeated measure analysis of variance (rmANOVA). Data for ROS, GSH, and cysteine did not pass normality and were log transformed. An rmANOVA with Bonferroni post hoc testing was used to analyze GSH, cysteine, ROS, SOD, GPx, and CoQ10. Paired *t*-tests were used to analyze CS activities. Analyses were performed using GraphPad Prism software version 8.2.0 (GraphPad Software San Diego, CA, USA). Significance was set at *p* < 0.05.

### 3.4. Muscle Proteomics

Proteomic analysis was performed on the 29- and 59-Day resting muscle samples obtained from the five geldings in the study using tandem mass tag 11-plex MS/MS quantification analysis at the Michigan State University Proteomics Core. Protein was extracted from muscle samples using a radioimmunoprecipitation lysis buffer and protease inhibitor and pelleted prior to submission. From each sample, 500 μg of protein was digested in trypsin with a filter-aided sample preparation protocol and spin ultrafiltration unit cutoff of 30,000 Da [28]. Reverse-phase C18 SepPaks were used to de-salt the resulting peptides (Waters Corporation, Milford, MA, USA) which were then dried by vacuum centrifugation. Peptide quantification was verified by colorimetric peptide concentration with 5 μL from each sample digest using a pierce BCA assay kit (Thermo Fisher Scientific, Waltham, MA, USA). Isobaric labeling, gel fractionation, and LC/MS/MS analysis were performed as previously described [22].

**Quantitative data analysis**: Scaffold Q+ (version Scaffold_4.9.0, Proteome Software Inc., Portland, OR, USA) was used to quantitate TMT Label Based Quantitation peptide and protein identifications. Peptide identifications were accepted if they could be established at greater than 95.0% probability by the Scaffold Local FDR algorithm. Protein identifications were accepted if they could be established at greater than 99.9% probability and contained at least two identified peptides. Protein probabilities were assigned by the Protein Prophet algorithm [29]. Proteins that contained similar peptides and could not be differentiated based on MS/MS analysis alone were grouped to satisfy the principles of parsimony. Proteins sharing significant peptide evidence were grouped into clusters. Channels were corrected by the matrix in all samples according to the algorithm described in i-Tracker [30]. Normalization was performed iteratively (across samples and spectra) on intensities, as described in *Statistical Analysis of Relative Labeled Mass Spectrometry Data from Complex Samples Using ANOVA* [31]. Medians were used for averaging. Spectra data were log-transformed, pruned of those matched to multiple proteins, and weighted by an adaptive intensity weighting algorithm. Of 108,665 spectra in the experiment at the given thresholds, 79,921 (74%) were included in quantitation. Differentially expressed proteins were determined by applying a permutation test with unadjusted significance level *p* < 0.003 corrected by Benjamini–Hochberg. Significant proteins were grouped according to their cellular functions.

## 4. Results

**Figure 1:**
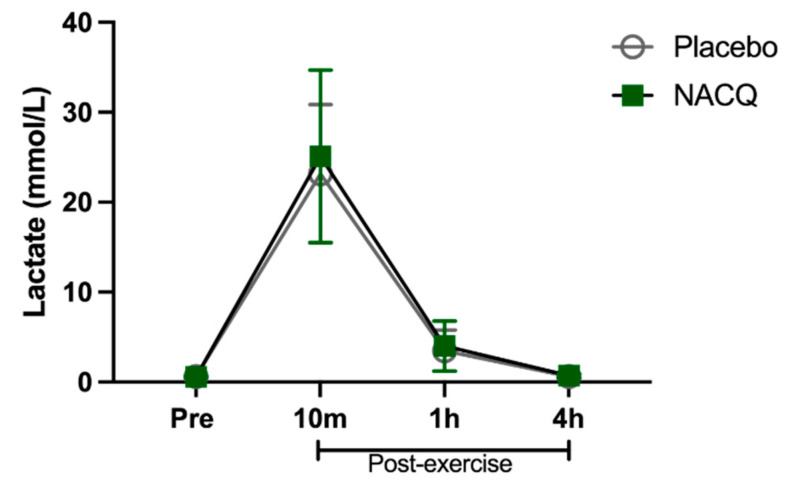
The concentration of plasma lactate in horses supplemented with NACQ compared to placebo before, during, and after an exercise test at a maximal gallop. Lactate concentrations were significantly higher 10 min post-exercise than all other timepoints (*p* < 0.0001) and were not significantly different between placebo and NACQ.

Exercise test, heart rates, and lactate concentrations: The maximum speed attained (NACQ 16.1 ± 0.7 m/s; placebo 16.1 ± 0.9 m/s) and maximum heart rate (NACQ 206 ± 8.34 bpm; placebo 207 ± 9.38 bpm) did not differ between NACQ and placebo (*p* = 0.69) or between Day 30 and Day 60 (*p* = 0.58). Plasma lactate concentrations were significantly higher (*p* < 0.0001) at 10 min post-exercise than all other timepoints and did not differ between NACQ and placebo (*p* = 0.68) (Figure 1).

Plasma CK and GGT activities increased significantly from rest to 4 h post-exercise (CK *p* < 0.001, GGT *p* = 0.008) and did not differ significantly between placebo and NACQ (CK *p* = 0.64, GGT *p* < 0.99) (Table 2).

### 4.1. Muscle Biochemistry

**Figure 2:**
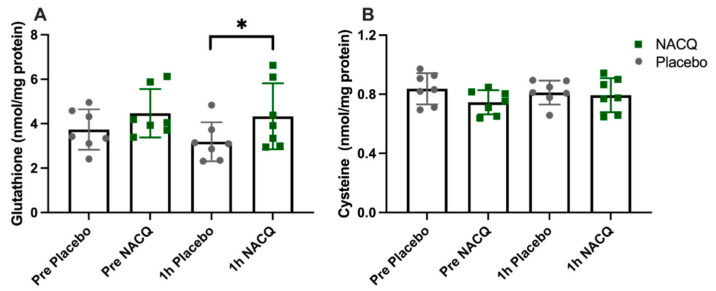
(**A**) Gluteal muscle glutathione concentrations in horses at rest and 1 h after exercise with NACQ (green) or placebo (grey) supplementation. The 1 h-post-exercise glutathione concentrations were significantly higher in NACQ horses than placebo (*p* = 0.022). (**B**) Muscle cysteine concentrations at rest and 1 h after exercise in horses supplemented with NACQ or placebo. Concentrations were not significantly different (*p* = 0.40). * *p* < 0.05 when compared with placebo group.

ROS, glutathione, and cysteine concentrations: The average coefficient of variance between replicates across all samples was 3.9%. There was no significant difference in muscle ROS between NACQ and placebo either before or after exercise (Table 3). ROS concentrations did not change significantly with exercise on either the NACQ or the placebo and showed wide individual variation. Muscle glutathione concentrations increased significantly by 36% after exercise (*p* = 0.022) on NACQ compared to placebo whereas cysteine concentrations did not differ significantly (*p* = 0.40) (Figure 2A,B).

**Figure 3:**
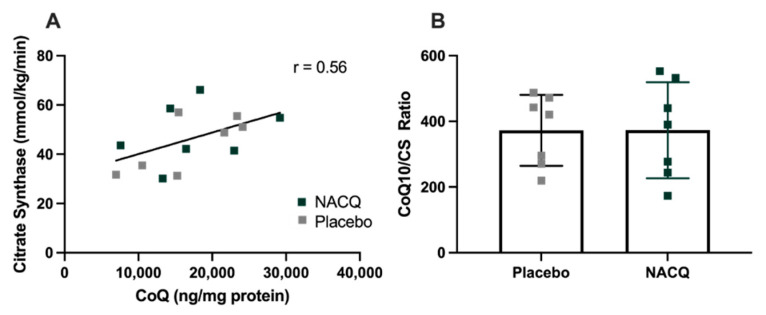
(**A**) Correlation between muscle coenzyme Q10 concentrations and CS activity in resting gluteal muscle samples (*R* = 0.56, *p* = 0.003). (**B**) The ratio of COQ10/CS in horses supplemented with NACQ or placebo. No significant differences were apparent (*p* = 0.99).

CoQ10 and citrate synthase: resting muscle CoQ10 concentrations did not differ significantly between NACQ and placebo or pre- and post-exercise samples. Considerable interindividual variation was found in muscle CoQ10 concentrations. CS activity did not differ between NACQ and placebo (*p* = 0.36) or between muscle samples obtained on Day 29 and Day 59 (*p* = 0.88). CoQ10 concentrations were significantly moderately correlated with CS activity (*r* = 0.56, *p* = 0.003) (Figure 3A). Muscle CoQ10 expressed as CoQ10 concentration/unit CS activity did not differ significantly between NACQ and placebo (*p* = 0.99) (Table 3, Figure 3B).

**Figure 4:**
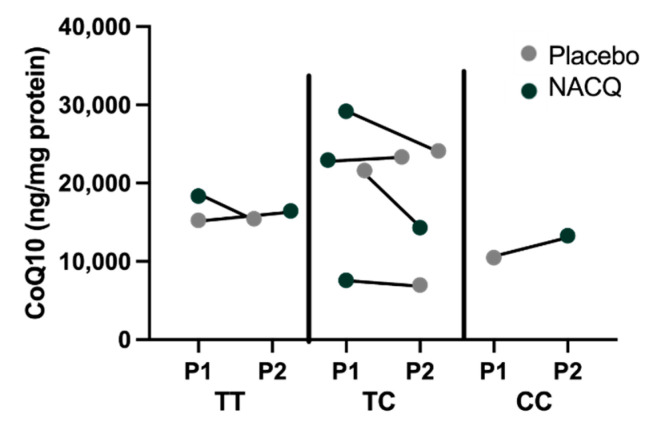
Myostatin genotypes and CoQ10 concentrations in horses on the placebo (grey) and on NACQ (green). The order of the points represents the order in which horses were fed the placebo or NACQ in the randomized block design.

Myostatin genotypes: one horse was homozygous for the g.66,493,737 C>T variant previously associated with a sprinter phenotype, two horses were the homozygous wild type previously associated with a stayer phenotype, and four horses were heterozygous [32]. There did not appear to be a particular pattern of CoQ10 concentrations or response to CoQ10 supplementation amongst the three genotypes although there were too few horses for a statistical analysis (Figure 4).

Glutathione peroxidase and superoxide dismutase activity: there was no significant difference in muscle glutathione peroxidase or superoxide dismutase activities in resting or post-exercise muscle between NACQ and placebo (Table 3).

### 4.2. Proteomics

**Figure 5:**
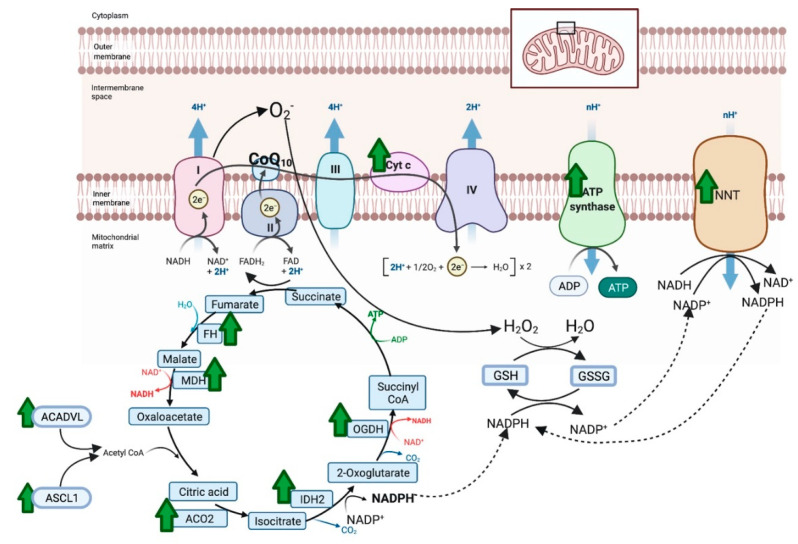
Proteins with significantly increased expression (green arrow) in the mitochondria of horses on NACQ compared to placebo. Horses with NACQ supplementation had upregulation of two cytochrome C subunits, one subunit of ATP synthase, reduced glutathione (GSH), oxidized glutathione (GSSG), NAD(P) transhydrogenase (NNT), fumarate hydratase (FH), malate dehydrogenase (MDH), 2-oxoglutarate dehydrogenase (OGDH), NADP-dependent isocitrate dehydrogenase (IDH2), aconitase (ACO2), Very Long-Chain Specific Acyl-CoA Dehydrogenase Mitochondrial (ACADVL), and Long-Chain-Fatty-Acid–CoA Ligase 1 Isoform X2 (ASCL1). Created with BioRender.com (accessed on 26 October 2021).

There were 387 unique total proteins identified in the proteomic dataset with 40 proteins differentially expressed between NACQ and placebo: 22 with increased expression and 18 decreased expression (Table 4). Proteins with increased expression largely localized to the mitochondrion and the sarcomeric Z disc with three appearing to be blood-borne (albumen, apolipoprotein A1, serum macroglobulin) (Table 4). Mitochondrial proteins included five proteins involved in the generation of NADH in the tricarboxylic acid cycle, three protein subunits in the electron transfer system, and three proteins involved in long-chain fatty-acid metabolism or transport (Table 4, Figure 5). The upregulated citric acid cycle proteins included two enzymes that produce succinyl CoA, isocitrate dehydrogenase (IDH2), and 2-oxogluterate dehydrogenase (OGDH). As well as ODGH, there was an additional upregulated protein in the mitochondrial inner membrane, NADP transhydrogenase (NNT), which generates nicotinamide adenine dinucleotide phosphate (NADPH) required to reduce oxidized glutathione. Upregulated proteins in the sarcomere were either components of the Z disc or a Z-disc-associated chaperone protein (CRAYB) that prevents protein misfolding. The sarcomeric Z-disc defines the lateral borders of the sarcomere and is important for mechanical stability of contractile filaments.

Proteins with decreased expression localized to the sarcoplasmic reticulum, the sarcomere, and the myoplasm (Table 4). This included -glutamate O-methyltransferase (ARMT1), which is involved in the synthesis of cysteine from methionine, fast-twitch 2X myosin (MYH1), the fast-twitch sarcoplasmic reticulum ATPase (ATP2A1), and nine glycolytic/gluconeogenic enzymes (Table 4).

Several antioxidant proteins were expressed in the proteomic dataset but were not differentially expressed, including mitochondrial superoxide dismutase [Mn], superoxide dismutase [Cu-Zn], catalase isoform X1, glutathione S-transferase P, cluster of glutathione S-transferase Mu 1, and mitochondrial thioredoxin-dependent peroxide reductase (Supplemental Table S1).

## 5. Discussion

The present study examined the impact of 30 d of supplementation of NACQ on non-enzymatic (glutathione, CoQ10) and enzymatic (glutathione-peroxidase and superoxide dismutase) antioxidants in skeletal muscle as well as the effect on the muscle proteome in fit Thoroughbred horses. Results showed that NACQ supplementation significantly enhanced post-exercise glutathione concentrations, enhanced proteins involved in reduction of oxidized glutathione, and enhanced proteins involved in mitochondrial oxidative energy metabolism and the Z disc while decreasing glycolytic enzyme and fast-twitch type 2X myosin protein expression.

Skeletal muscle glutathione concentrations were measured previously in several species with wide-ranging results depending on the method of analysis (Table S1) [33,34,35,36,37,38,39,40,41]. When predominantly fast-twitch muscle such as the human vastus lateralis or “quadriceps” (~60% fast-twitch muscle fiber; glutathione 1.2–1.4 nmol/mg wet weight) [42] were compared to Thoroughbred gluteal muscle (~80% fast-twitch muscle fibers 0.85 nmol/kg wet weight) [43] measured by HPLC, horses appeared to have approximately 40% lower glutathione concentrations than humans. This could be in part be due to the higher fast-twitch fiber type composition of horse gluteal muscle compared to human vastus lateralis or, intriguingly, equine muscle could have a lesser capacity to synthesize glutathione or an enhanced turnover of glutathione with exercise.

Glutathione concentrations were significantly higher (↑ 35%) 1 h post-exercise in horses on NACQ compared to placebo but did not differ significantly between treatments at rest. This agrees with a study of human athletes taking 60 mg/kg oral NAC that showed a significant increase in glutathione concentrations (~14%) 2 h after, but not before, exercise in vastus lateralis muscle on supplementation [36]. The reason for the post-exercise increase in glutathione could be stimulation of enzymes involved in glutathione synthesis by exercise combined with increased availability of muscle cysteine [41]. Oral NAC provides a readily absorbable form of cysteine that liberates endogenous, protein-bound cysteine in plasma [44] and enhances transport of cysteine into muscle cells by the gamma-glutamyl cycle [45,46]. A measurable increase in skeletal muscle cysteine concentrations was not detected on NACQ, which could have been due to rapid incorporation of soluble cysteine into thiol-based antioxidants and other proteins. After exercise, gamma-glutamyl transferase had significantly increased serum activity in our horses, which could have facilitated rapid cysteine transport into muscle and subsequent synthesis of glutathione in the 1 h-post-exercise samples. A potential reduction in synthesis of cysteine from methionine in horses on NACQ was supported by the finding that glutamate O-methyltransferase (ARMT1) had decreased protein expression in NACQ- versus placebo-supplemented muscle. ARMT1 is one of the enzymes required to synthesize cysteine from methionine [47].

An enhanced ability to shuttle glutathione into mitochondria and to reduce oxidized glutathione in horses on the NACQ supplement was supported by proteomic data. Fumerate (FH) and 2-oxoglutarate dehydrogenase (OGDH), which produce malate/2-oxoglutarate for the SLC25A mitochondrial glutathione shuttle, had increased expression on NACQ vs. placebo (Figure 5). Additionally, isocitrate dehydrogenase (IDH2) and NADP+ transporter (NNT), which generate NADPH to reduce glutathione, also had increased expression [48] (Figure 5). Taken together, the results of our study support the ability of NACQ supplementation to enhance post-exercise muscle glutathione concentrations, likely through increased muscle cysteine availability, and also the potential ability of NACQ to enhance the capacity of muscle to reduce oxidized glutathione.

Enzymatic antioxidants identified either in the proteome or by measuring enzymatic activities in muscle samples were not altered by NACQ supplementation. The ability of a number of these antioxidants to continue functioning as reducing agents, however, could be indirectly enhanced by supplementation with NACQ by providing a ready supply of glutathione, which is reciprocally oxidized in the process of reducing oxidized glutathione peroxidase or thioredoxins and peroxiredoxins [49].

Coenzyme Q10 was the additional antioxidant in the NACQ supplement. Similar to studies in healthy humans, we did not find a significant increase in muscle CoQ10 concentrations after CoQ10 supplementation. Neither a 14 d trial with an oral fast-melt CoQ10 formulation (manufacture’s recommended dose 100 mg/d) [50] nor a 28 d trial using 150 mg oral capsule supplementation produced an increase in muscle CoQ10 in healthy subjects [51]. Muscle CoQ10 concentrations were shown, however, to increase with supplementation in humans with deficiencies in enzymes required to synthesize CoQ10 [14]. Concentrations of muscle CoQ10 in our healthy horses were found to be similar to those measured in healthy humans using a similar MS/MS analysis [52]. The absence of an increase in CoQ10 muscle concentrations in supplemented horses during the present study could have arisen from inhibition of extraneous muscle CoQ10 absorption due to optimal endogenous muscle concentrations.

There have been previous equine studies measuring the effect of CoQ10 supplementation on plasma CoQ10 concentrations [15,53,54]. Two of the studies utilized oral CoQ10 supplementation (HydroQ-Sorb; GelTec/Tishcon Corp., Westbury, NY), with one study feeding 800 mg/d for 60 d [53] and the other utilizing two different doses of 1.9 g and 3.4 g as acute doses to measure their effect on exercise [15]. The third study utilized supplementation with both 800 mg CoQ10 (Vital Paste 7.5%, Valens Int. d. o. o., Šenčur, Slovenia) and 1.8 IU/kg BW/d Vitamin E (d-α-tocopherol acetate; natural vitamin E-oil, Natural Wealth, Bohemia, NY, USA) for 14 d [54]. The first two studies found an increase in plasma CoQ10 concentrations [15,53]; however, this was attenuated with exercise in the second study [15], and the third study found an increase in plasma CoQ10 when supplemented with both CoQ10 and Vitamin E [54]. It has not been established whether an increase in plasma CoQ10 concentration correlates with an increase in muscle CoQ10 concentrations.

Our results are in contrast to a previous equine study in which six fit Thoroughbred horses were supplemented with 1 g of ubiquinol (Recovery Sport, Anlon Nutrition, Kilcullen, Ireland) for 3 wk using a cross-over design [17]. The previous study found a significant increase in muscle CoQ10 concentrations, which was largely driven by two out of six individual horses. Muscle CoQ10 concentrations in the previous study were reported to be similar to concentrations in human muscle measured using an HPLC method. Notably, CoQ10 concentrations measured by HPLC in the previous equine study were approximately 100× lower than those measured in our study using an MS/MS method [17]. The differences in results between the two equine studies could be due to methodological differences, differences in gastrointestinal absorption of the different CoQ10 supplements, or the small number of horses and the large individual variability in both CoQ10 concentrations and responses to supplementation. Unfortunately, serial blood samples coordinated with oral supplementation were not obtained in the present or the previous study of equine muscle CoQ10 concentrations [17].

It has been suggested that wide individual variation of CoQ10 concentrations in horses could be linked to myostatin genotypes based on a study that inferred CoQ10 concentrations from mitochondrial complex I + III and complex II + III activities [55]. Although the number of horses with diverse genotypes in our study was small, visual inspection of the data did not identify a relationship between myostatin genotype and either muscle CoQ10 concentrations measured by MS/MS or responses to CoQ10 supplementation (Figure 4).

The near-maximal exercise tests used in our study were performed on a standard racetrack and appeared highly repeatable as there were no differences in maximal speed, maximal heart rate, or plasma lactate concentrations between the first and second exercise tests. Our cross-over design, however, did not allow us to evaluate any potential long-term benefit of NACQ supplementation on athletic performance, nor did the use of combined NAC and CoQ10 supplementation allow us to differentiate alterations caused by NAC from those caused by CoQ10. The cross-over design did mitigate any potential effects of training in this study, as confirmed by muscle CS activity, which was not significantly different between Days 29 and 59 in our study. In a field setting, volatile Thoroughbreds require muscle biopsies to be obtained following a cool-down period and not immediately after exercise. At one hour after exercise, an increase in ROS was not identified and no differences were found in ROS between NACQ and placebo before or after exercise. Unfortunately, our study could not ascertain whether or not high amounts of ROS were generated during exercise without further serial biopsies. A previous study found an increase in plasma malondialdehyde (MDA) activity after exercise in untrained horses in both the placebo and the CoQ10 groups, suggesting that ROS is generated during exercise [54]. The increase in MDA was attenuated by additional supplementation with Vitamin E [54].

The TMT proteomic analysis used in the present study evaluated the relative abundance of muscle proteins with NACQ supplementation relative to the placebo. The study assessed comparisons within the same individual, minimizing intra-individual variability common with repeated measures. The log_2_FC differences identified were relatively small. However, the biological relevance of the changes was supported by the fact that proteins with related functions had similar patterns of differences. The general tendency in the proteomic analysis was for an increase in proteins involved in oxidative metabolism and a decrease in proteins involved in glycolytic metabolism, indicating a relative shift toward a more oxidative phenotype. This shift was not sufficient to produce an increase in CS activity, which is often used as a marker for enhanced mitochondrial volume density in horses [56]. The functional impact of the small, yet significant, alterations in the muscle proteome were not assessed in the present study.

The ability of the supplement to enhance glutathione, as well as redox-sensitive chaperone proteins such as CRYAB, has applications for diseases such as myofibrillar myopathy (MFM) [57,58]. Arabian horses with MFM have Z-disc disruption and alterations in pathways of cysteine synthesis with decreased expression of another thiol-based antioxidant, peroxiredoxin, that can be reduced by glutathione [22]. Warmblood horses with MFM have alterations in Z-disc proteins, enrichment of pathways of oxidative stress, and markedly increased expression of the gene *CHAC1*, which encodes an enzyme that degrades glutathione [59]. The results of the present study suggest that evaluating the impact of NACQ supplementation on horses with MFM is warranted. Concerns have been raised in human medicine about antioxidant supplementation impairing training adaptations mediated by ROS signaling [7]. None of the horses in the present study, however, had any impairment of exercise responses while on the NACQ supplement and there was no impact on CS activity, an indicator of training response.

## 6. Conclusions

In conclusion, supplementation of fit Thoroughbred horses with N-acetyl cysteine and CoQ10 for 30 d appears to impact muscle redox status without any evident detrimental effects on performance. Muscle glutathione concentrations after intense exercise were significantly increased and expression of proteins involved in the uptake of glutathione into mitochondria, and the reduction of oxidized glutathione, were enhanced.

## Figures and Tables

**Table 1:** Guaranteed analysis of the OBS Racing Blend concentrate provided to the horses throughout the study at 2.1 kg/500 kg body weight.

Nutrient	Amount
Crude Protein (Min)	12.0%
Lysine (Min)	0.7%
Crude Fat (Min)	8.0%
Crude Fiber (Max)	9.0%
Calcium (Min)	0.6%
Calcium (Max)	0.7%
Phosphorus (Min)	0.5%
Selenium (Min)	0.5 ppm
Zinc (Min)	145 ppm
Vitamin A (Min)	11,000 IU/kg
Vitamin D (Min)	1100 IU/kg
Vitamin E (Min)	211 IU/kg

**Table 2:** Plasma gamma-glutamyl transferase (GGT) and creatine kinase (CK) activity in NACQ- and placebo-supplemented horses. Differences in superscripts a and b indicate significant differences with supplementation or exercise. There were no significant effects of NACQ/placebo supplementation, however there was a significant effect of exercise on GGT (*p* = 0.008 and CK activities (*p* < 0.001).

	GGT (U/L)	CK (U/L)
	Mean ± Sd	Mean ± Sd
Placebo Pre	18.7 ± 6.0 ^a^	190.0 ± 44.3 ^a^
Placebo 4 h Post	25.0 ± 8.0 ^b^	446.1 ± 171.2 ^b^
NACQ Pre	20.0 ± 7.2 ^a^	278.4 ± 151.4 ^a^
NACQ 4 h Post	22.6 ± 5.7 ^b^	387.4 ± 146.2 ^b^

**Table 3:** Skeletal muscle reactive oxygen species (ROS), coenzyme Q10 (CoQ10) concentrations, and activities of glutathione peroxidase (GPx) and superoxide dismutase (SOD) in NACQ- and placebo-supplemented horses. Differences in superscripts indicate significant differences with supplementation or exercise. There were no significant effects of exercise or NACQ/placebo supplementation.

	Supplement	Rest	1 h Post-Exercise	*p* Value
ROS	NACQ	16.51 ± 6.14 ^a^	14.68 ± 5.60 ^a^	
(μM H_2_O_2_ equivalence)	Placebo	13.81 ± 4.83 ^a^	17.55 ± 5.00 ^a^	0.4779
CoQ10	NACQ	7090 ± 2898 ^a^	7653 ± 3632 ^a^	
(ng/mg protein)	Placebo	7679 ± 2442 ^a^	8560 ± 416 ^a^	0.8352
GPx	NACQ	13.81 ± 4.83 ^a^	17.55 ± 5.00 ^a^	
(mmol/min/mg protein)	Placebo	25.53 ± 5.08 ^a^	21.52 ± 5.53 ^a^	0.2065
SOD	NACQ	7.178 ± 1.238 ^a^	6.604 ± 0.792 ^a^	
(U/mg protein)	Placebo	7.377 ± 1.551 ^a^	7.390 ± 1.187 ^a^	0.6191

**Table 4:** The gene identification for a protein, protein name, log_2_ fold change, and *p* value adjusted for multiple test corrections in five geldings supplemented with NACQ compared to placebo.

Gene ID	Protein Name	Adjusted *p* Value	Log_2_ Fold Change
**Increased expression**			
MB	Myoglobin	1.90 × 10^−3^	0.22
**Mitochondria**			
	**TCA cycle**		
IDH2	Isocitrate Dehydrogenase [NADP] Mitochondrial	2.10 × 10^−4^	0.12
ACO2	Aconitate Hydratase Mitochondrial	5.00 × 10^−4^	0.11
MDH2	Malate Dehydrogenase Mitochondrial	4.00 × 10^−3^	0.11
FH	Fumarate Hydratase Mitochondrial	2.00 × 10^−3^	0.11
OGDH	2-Oxogluterate Dehydrogenase Mitochondrial Isoform X3	8.70 × 10^−4^	0.09
	**Electron Transfer System**		
COX5A	Cytochrome C Oxidase Subunit 5A Mitochondrial	8.90 × 10^−4^	0.18
COX4I1	Cytochrome C Oxidase Subunit 4 Isoform 1 Mitochondrial	5.00 × 10^−4^	0.13
ATP5F1B	ATP Synthase Subunit beta Mitochondrial	1.00 × 10^−4^	0.10
	**Fat metabolism**		
ACADVL	Very Long-Chain Specific Acyl-CoA Dehydrogenase Mitochondrial	1.00 × 10^−3^	0.11
ASCL1	Long-Chain-Fatty-Acid–CoA Ligase 1 Isoform X2	2.70 × 10^−3^	0.10
FABP3	Fatty Acid-Binding protein Heart	1.00 × 10^−3^	0.27
	**Other**		
NNT	NAD(P) Transhydrogenase Mitochondrial Isoform X1	3.00 × 10^−3^	0.07
VDAC2	Voltage-Dependent Anion-Selective Channel Protein 2	2.00 × 10^−3^	0.14
**Sarcomere Proteins**			
**Z disc**			
CRYAB	Alpha-Crystallin B Chain	1.00 × 10^−4^	0.28
ACTN2	Alpha-Actinin-2 Isoform X1	1.00 × 10^−4^	0.16
FLNC	Filamin-C Isoform X1	1.30 × 10^−4^	0.12
PDLIM5	PDZ and LIM Domain Protein 5 Isoform X9	7.20 × 10^−4^	0.12
**Myosin**			
MYBPC1	Myosin-Binding Protein C Slow-Type Isoform X2	1.00 × 10^−4^	0.11
**Blood Proteins**			
ALB	Serum Albumin Precursor	1.00 × 10^−4^	0.38
APOA1	Apolipoprotein A-I	1.00 × 10^−3^	0.34
LOC100061692	Alpha-2 Macroglobulin	3.50 × 10^−3^	0.22
**Decreased expression**			
**Cysteine Synthesis**			
ARMT1	Protein-Glutamate O-Methyltransferase	7.30 × 10^−4^	−0.40
**Glycolysis/Gluconeogenesis**			
PFKM	ATP-Dependent 6-Phosphofructokinase Muscle Type Isoform X3	2.00 × 10^−3^	−0.07
PKM	Pyruvate Kinase PKM Isoform M1	1.00 × 10^−4^	−0.10
LDHA	L-lactate Dehydrogenase A Chain	1.80 × 10^−4^	−0.10
FBP2	Fructose -1,6-bisphosphatase isozyme 2	4.00 × 10^−3^	−0.11
GPI	Glucose-6-Phosphate Isomerase	1.00 × 10^−4^	−0.14
PHKA1	Phosphorylase B Kinase Regulatory Subunit alpha Skeletal Muscle Isoform X1	1.00 × 10^−3^	−0.14
PHKB	Phosphorylase B Kinase Regulatory Subunit beta	2.00 × 10^−3^	−0.14
ENO3	Beta-Enolase	1.00 × 10^−4^	−0.15
PGM1	Phosphoglucomutase-1 Isoform X2	1.00 × 10^−4^	−0.15
**Sarcomere**			
MYOM1	Myomesin-1 Isoform X4	1.00 × 10^−3^	−0.08
MYOM2	Myomesin-2 Isoform X3	1.00 × 10^−4^	−0.12
MYH1	Myosin-1 Isoform X1	2.70 × 10^−4^	−0.11
MYBPC2	Myosin-Binding Protein C Fast-Type	1.00 × 10^−4^	−0.17
MYLK2	Myosin Light Chain Kinase 2 Skeletal/Cardiac Muscle	2.50 × 10^−3^	−0.20
**Calcium Regulation**			
SRL	Sarcalumenin Isoform X1	1.00 × 10^−3^	−0.09
ATP2A1	Sarcoplasmic/Endoplasmic Reticulum Calcium ATPase 1 Isoform X1	1.00 × 10^−4^	−0.17
ANXA6	Annexin A6 Isoform X1	1.00 × 10^−4^	−0.13

## Data Availability

Data is contained within the article or Supplementary Materials.

## Supplementary Materials

The following are available online at www.mdpi.com/article/10.3390/antiox10111739/s1, Table S1: Glutathione concentrations in skeletal muscle across different species.
