# Supplementary material for: The Impact of N-Acetyl Cysteine and Coenzyme Q10 Supplementation on Skeletal Muscle Antioxidants and Proteome in Fit Thoroughbred Horses
Source: Antioxidants (Basel). 2021 Oct 30;10(11):1739. doi: 10.3390/antiox10111739 (PMC8615093; doi:10.3390/antiox10111739)
Supplement: Supplementary file 1 [file antioxidants-10-01739-s001.zip › antioxidants-1396794-supplementary.pdf]

Table S1. Glutathione concentrations in skeletal muscle across different species. Each study is presented with species it was measured in, analysis type performed, exercise type, muscle type, and concentrations in their respective units. Also provided are concentrations from this study for comparison in both nmol/mg protein as shown previously as well as an estimation of concentrations in nmol/mg tissue.

Study	Species	Analysis	Exercise	Muscle	[GSH]	
Present study	Horse	HPLC LC/MS/MS	Pre	Gluteus Medius	3.74 ± 0.91 nmol/mg protein	mean ± SD
			1h Post		3.19 ± 0.88 nmol/mg protein	
			Pre		~1.00 ± 0.24 nmol/mg WW	
			1h Post		~0.85 ± 0.23 nmol/mg WW	
Martensson and Meister, 1989 [32]	Mice	glutathione disulfide reductase-5,5'-dithiobis(2-nitrobenzoate) recycling method	No	Quadriceps	0.77 ± 0.53 nmol/mg WW	mean ± SD
				Skeletal muscle mitochondria	5.70 ± 0.30 nmol/mg protein	
				Heart	1.15 ± 0.071 nmol/mg WW	
				Heart mitochondria	11.50 ± 0.40 nmol/mg protein	
Ji *et al.,* 1992 [33]	Rat	HPLC method	Pre	Superficial Vastus Lateralis	0.55 ± 0.05 nmol/mg WW	mean ± SEM
				Deep Vastus Lateralis	1.5 ± 0.02 nmol/mg WW	
				Soleus	3.2 ± 0.2 nmol/mg WW	
			25m/min 5% grade	Superficial Vastus Lateralis	0.45 ± 0.05 nmol/mg WW	
				Deep Vastus Lateralis	1.7 ± 0.3 nmol/mg WW	
				Soleus	3.5 ± 0.2 nmol/mg WW	

			25m/min 10% grade	Superficial Vastus Lateralis	0.6 ± 0.05 nmol/mg WW	
				Deep Vastus Lateralis	1.8 ± 0.2 nmol/mg WW	
				Soleus	3.2 ± 0.2 nmol/mg WW	
Dam *et al.,* 2012 [34]	Rats	HPLC and detected at 350nm	No	Quadriceps	1.40 ± 0.03 nmol/mg WW	mean ± SEM
Morin *et al.,* 2019 [35]	Gunea Pig	Capillary electrophoresis/UV	No	Gastrocnemius	10 ± 2 nmol/mg protein	mean ± SD
Marin *et al.,* 1993 [36]	Dog	Spectophotometrically at 412nm	Untrained	Gastrocnemius	1.56 ± 0.17 nmol/mg WW	mean ± SD
			Trained		2.22 ± 0.24 nmol/mg WW	
Luo *et al.,* 1996 [37]	Human	HPLC seperation with fluorescent detection at excitation 394nm and emission 480 nm	No	Quadriceps femoris	1.43 ± 0.17 nmol/mg WW	mean ± SEM
Hammarqvist *et al.,* 1997 [38]	Human	HPLC seperation with fluorescent detection at excitation 394nm and emission 480 nm	No	Vastus Lateralis	1.42 ± 0.041 nmol/mg WW	mean ± SD
Hammarqvist *et al.,* 2005 [39]	Human	HPLC seperation with fluorescent detection at excitation 394nm and emission 480 nm	No	Quadriceps femoris	1.21 ± 0.15 nmol/mg WW	mean ± SD
Michailidis *et al.,* 2013 [40]	Human	Spectophotometrically at 375nm	Pre	Vastus Lateralis	55.0 ± 3.00 nmol/mg protein	mean ± SD
			2 h post		43.0 ± 3.00 nmol/mg protein	
			48 h post		34.0 ± 2.00 nmol/mg protein	

			8 d post		47.0 ± 3.00 nmol/mg protein	
